# The Standardization of Hospital-Acquired Infection Rates Using Prediction Models in Iran: Observational Study of National Nosocomial Infection Registry Data

**DOI:** 10.2196/33296

**Published:** 2021-12-07

**Authors:** Neda Izadi, Koorosh Etemad, Yadollah Mehrabi, Babak Eshrati, Seyed Saeed Hashemi Nazari

**Affiliations:** 1 Department of Epidemiology School of Public Health and Safety Shahid Beheshti University of Medical Sciences Tehran Iran; 2 Department of Social Medicine School of Medicine Iran University of Medical Sciences Tehran Iran; 3 Prevention of Cardiovascular Disease Research Center Department of Epidemiology School of Public Health and Safety, Shahid Beheshti University of Medical Sciences Tehran Iran

**Keywords:** hospital-acquired infections, standardized infection ratio, prediction model, Iran

## Abstract

**Background:**

Many factors contribute to the spreading of hospital-acquired infections (HAIs).

**Objective:**

This study aimed to standardize the HAI rate using prediction models in Iran based on the National Healthcare Safety Network (NHSN) method.

**Methods:**

In this study, the Iranian nosocomial infections surveillance system (INIS) was used to gather data on patients with HAIs (126,314 infections). In addition, the hospital statistics and information system (AVAB) was used to collect data on hospital characteristics. First, well-performing hospitals, including 357 hospitals from all over the country, were selected. Data were randomly split into training (70%) and testing (30%) sets. Finally, the standardized infection ratio (SIR) and the corrected SIR were calculated for the HAIs.

**Results:**

The mean age of the 100,110 patients with an HAI was 40.02 (SD 23.56) years. The corrected SIRs based on the observed and predicted infections for respiratory tract infections (RTIs), urinary tract infections (UTIs), surgical site infections (SSIs), and bloodstream infections (BSIs) were 0.03 (95% CI 0-0.09), 1.02 (95% CI 0.95-1.09), 0.93 (95% CI 0.85-1.007), and 0.91 (95% CI 0.54-1.28), respectively. Moreover, the corrected SIRs for RTIs in the infectious disease, burn, obstetrics and gynecology, and internal medicine wards; UTIs in the burn, infectious disease, internal medicine, and intensive care unit wards; SSIs in the burn and infectious disease wards; and BSIs in most wards were >1, indicating that more HAIs were observed than expected.

**Conclusions:**

The results of this study can help to promote preventive measures based on scientific evidence. They can also lead to the continuous improvement of the monitoring system by collecting and systematically analyzing data on HAIs and encourage the hospitals to better control their infection rates by establishing a benchmarking system.

## Introduction

Many factors contribute to the spreading of hospital-acquired infections (HAIs), and controlling nosocomial infections is now a global priority. Because accurate measurements are needed to improve any situation, infection control measures must first include accurate determination of the infection incidence at the hospital level [1]. On the other hand, statistics in low- and middle-income countries show that the exact rate of HAIs in such countries depends on 2 groups of factors: (1) factors related to the hospital, such as the hospital bed size (number of beds), the grade of the referral hospital, whether the hospital is teaching or nonteaching, the presence or absence of monitoring programs, the ward types, the facilities, and the adequacy of financial resources for such care programs, and (2) factors beyond the hospital’s control, such as the age and gender of patients [2-4]. Therefore, it is necessary to determine the rate of HAIs by adjusting and considering these variables and to obtain a suitable statistical model to calculate these rates.

On the other hand, environmental and sociocultural factors also contribute to rate of HAIs. In addition, the relation between HAI rates and the socioeconomic level showed that a lower country socioeconomic level was correlated with a higher infection risk [5,6]. The environmental factors include contaminated air-conditioning systems and the physical layout of the facility, and factors related to cultural issues include lack of or poor hand hygiene practice and awareness by health care workers and lack of use of sterile methods [6,7].

In the United States, the Centers for Disease Control and Prevention (CDC) uses the National Nosocomial Infections Surveillance (NNIS) risk index to determine the incidence of infections, especially for surgical site infections (SSIs) [8]. Despite the use of this risk index in many countries, the NNIS risk index has several drawbacks, including the following: using only 4 factors (including the American Society of Anesthesiologists score, wound class, procedure duration, and endoscope use) to calculate the risk index and its inadequacy, considering all variables as binary variables and not quantitative variables, and considering the same weights for different procedures and their inaccuracy (different weights are needed). For this reason, in 2009, the United States National Healthcare Safety Network (NHSN) introduced a new statistical model to replace the NNIS risk index to estimate the expected infection incidence for all infections, which was then used as a denominator in the new measurement: the standardized infection ratio (SIR) based on a statistical model [9,10]. The use of a statistical model–based SIR solved the problems of utilizing an SIR based on the conventional risk index in the United States and significantly improved international comparisons in this field [11]. The SIR is a summary measure used to track HAIs at a national, state, or local level over time. This measure compares the actual number of reported HAIs with the number that would be predicted, adjusting for the factors that are associated with differences in the infection incidence. By calculating the SIR, the relevant and significant variables for each hospital are adjusted.

In Iran, the Center for Disease Control and Prevention of the Ministry of Health and Medical Education is responsible for establishing a hospital infection surveillance system for regular and continuous data collection related to nosocomial infections, analysis, publication of periodic reports, and providing feedback. However, calculating only a crude rate without adjusting for the variables that affect HAIs and using this rate for planning and policy in this field may not be very efficient. Therefore, this study aimed to standardize the HAI rate using prediction models in Iran based on the NHSN method.

## Methods

### Surveillance System

In 2010, a surveillance system for infectious diseases was created in Iran, and all hospitals were requested to record information about HAIs in the hospital. The Iranian Nosocomial Infections Surveillance System (INIS) was revised in 2017. In 2018, it collected data from 863 hospitals in all the provinces of Iran and recorded different types of information. INIS uses the CDC/NHSN definitions to report all infections. In this study, INIS (126,314 patients with HAIs in 2018) and the hospital statistics and information system (AVAB), a web-based system for monitoring and evaluation of different hospitals (ie, data on 942 hospitals in Iran in 2018), were used to standardize the HAI rate. With the help of these systems and their linkage, we have access to data on the observed number of HAIs in different hospitals during the year of the study. More details about the data source, linkage of the data source, and national standard for hospital performance indicators (ie, average length of stay [LOS], bed occupancy rate [BOR], bed turnover [BTO] rate, death-to-bedridden ratio, and Pabon Lasso model) are available elsewhere [12,13].

### Ethics Approval and Consent to Participate

All procedures performed in this study were approved by the ethical committee of the National Institute for Medical Research Development (IR.NIMAD.REC.1399.074). All methods were carried out in accordance with relevant guidelines and regulations.

### Variable Selection

In this study, there were about 40 variables for the prediction model. Using univariable regression analysis (*P*<.20) and expert opinions, the important variables (24 variables) for the prediction model were selected: type of ward, hospital affiliation, type of hospital, hospital expertise, accreditation, number of active beds, average LOS, BTO rate, nurse-to-hospital bed ratio, number of devices used daily in each ward (device-day), number of catheters used daily in each ward (catheter-day), number of ventilators used daily in each ward (ventilator-day), mean age, length of hospitalization until infection, duration of hospitalization, male-to-female patient ratio, number of deaths in each ward, number of device-related infections, number of ventilator-related infections, number of catheter-related infections, BOR, surgery-to-surgery bed ratio, death-to-bedridden ratio, ventilator-day to catheter-day ratio.

### Well-Performing Hospitals in Terms of the Accreditation Degree and Reporting of HAIs for Modeling and Obtaining Coefficients for SIR Calculation

To develop a model that identifies the infection predictors in a normal hospital, first, well-performing hospitals were defined as follows: (1) hospitals with excellent and first grade “accreditation” and (2) hospitals with an HAI rate of 4%-15%.

Considering all the aforementioned criteria, the number of records decreased to 687. Missing items were also removed for the variable “surgery-to-surgery bed ratio” (23 records).

For the number of expected (predicted) infections (denominator of the SIR indicator), first, well-performing hospitals (with similar distribution of hospital expertise, type of ward, type of hospital, and affiliation compared with the total data) were selected, including 357 hospitals from 31 provinces and 54 universities in the country (664 records). Poisson regression analysis was performed on the data, and over/underdispersion was examined in the model. Then, due to overdispersion (μ<σ^2^) and because there was an excess of zero counts, generalized negative binomial and hurdle negative binomial regression analyses were performed on the data. Also, the “number of hospitalizations” logarithm was considered as an offset for the model. The previously mentioned predictors were entered into the model. Then, using the Akaike information criterion and both directions of the stepwise method (forward/backward), the candidate variables for the final models were determined.

### Model Validation

In this study, the data were randomly split into training (70%) and testing (30%) data sets. The final model for each type of HAI was developed on the training data, and then, the validity of the models was measured on the testing data using “pseudo R^2^” metrics including:



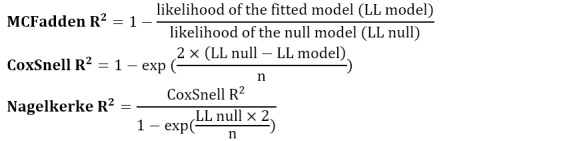



In addition, the model results from the training, test, and total data sets were compared based on the prediction error, which is the mean square error (MSE):



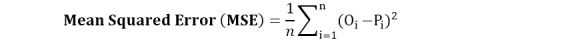



where O is observed and P is predicted.

Finally, the root mean square error (RMSE) was obtained by taking the root of the model prediction error value, and the final models were extracted to calculate the SIR using the lowest value of RMSE.

### SIR

This ratio is a summary measure that compares the observed number of HAIs and the number that would be predicted, adjusting for several risk factors that are associated with differences in the infection incidence. In other words, an SIR greater than 1.0 means that the observed number of HAIs is more than predicted; conversely, an SIR less than 1.0 indicates that fewer HAIs were observed than predicted.



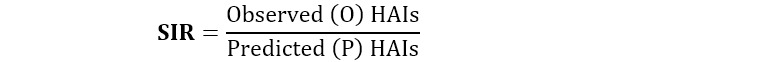



For each type of infection, the regression model on the total data (3449 records) was updated using regression coefficients of the model based on the data from well-performing hospitals (664 records), and the number of infections was predicted. Then, the SIR was calculated for the HAIs with different variables in Iran using the INIS data as the observed number of HAIs.

### Corrected SIR

**Figure 1:**
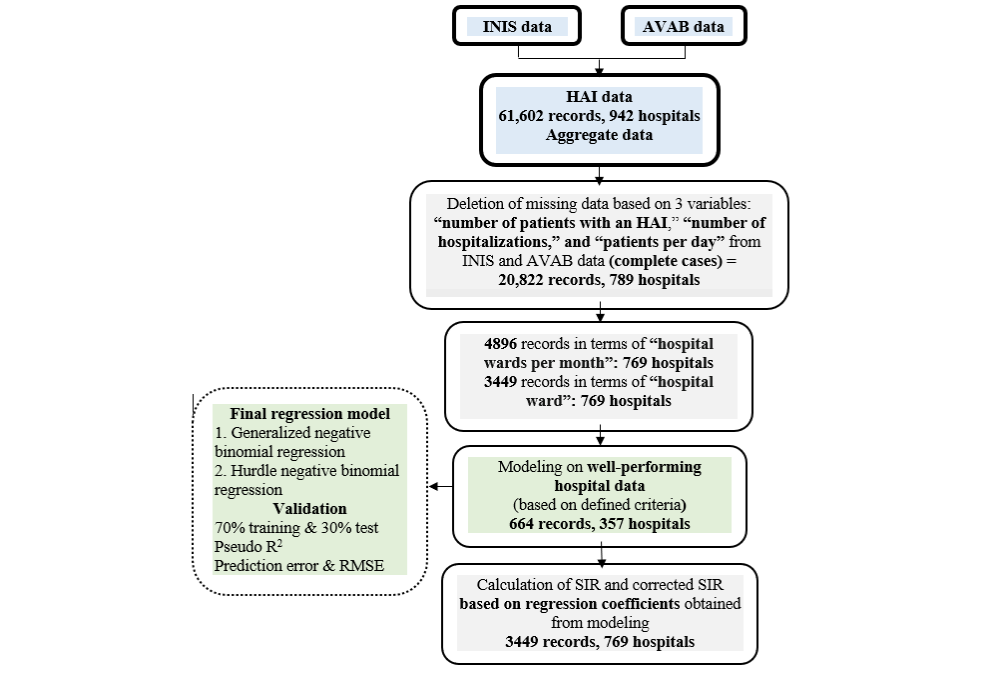
Data preparation steps and calculation of standardized infection ratio (SIR). AVAB: hospital statistics and information system; HAI: hospital-acquired infection; INIS: Iranian Nosocomial Infections Surveillance System; RMSE: root mean square error.

To calculate the correction factor (CF), linear regression analysis was used (no constant). The number of observed as the outcome variable (y) and the number of predicted (expected) obtained from the regression model for each infection from the data of well-performing hospitals (664 records) were defined as independent variables (x), and the coefficient obtained in the linear regression analysis was considered the CF. In the next step, the CF was multiplied by the number of expected (predicted) infections for the total data (3449 records). Finally, the corrected SIR was calculated (Figure 1).

The corrected SIR was calculated as follows: CF × number of expected (predicted) infections for the total data (3449 records).

## Results

The mean age of the 100,110 patients with an HAI was 40.02 (SD 23.56) years. The median hospitalization length until infection and hospital LOS were 6.97 (Q1-Q3 2.67-12.6) days and 15.2 (Q1-Q3 7.75-24.9) days, respectively.

### Model Development and Performance Measurement

The most important predictor variables in the training and test data and on well-performing hospital data were the type of ward, hospital affiliation, mean age of patients, male-to-female patient ratio, number of device-related infections, number of catheter-related infections, surgery-to-surgery bed ratio, death-to-bedridden ratio, and ventilator-day to catheter-day ratio. Table 1 shows the Cox and Snell R^2^, Nagelkerke R^2^, and RMSE values in the training, test, and total data sets for each type of infection.

**Table 1:** Performance measures for the hospital-acquired infection prediction model on the training, test, and total data sets from well-performing hospitals.

Performance measures			Training data (n=466)		Test data (n=198)		Total data (n=664)
**RTI^a^**							
	Pseudo R^2^: McFadden	0.19		0.22		-^b^	
	Pseudo R^2^: Cox and Snell (maximum likelihood)	0.74		0.79		-	
	Pseudo R^2^: Nagelkerke (Cragg and Uhler)	0.74		0.79		-	
	Prediction error (MSE^c^)	344.47		466.99		354.57	
	RMSE^d^	18.56		21.61		18.83	
**UTI^e^**							
	Pseudo R^2^: McFadden	0.20		0.19		-	
	Pseudo R^2^: Cox and Snell (maximum likelihood)	0.73		0.72		-	
	Pseudo R^2^: Nagelkerke (Cragg and Uhler)	0.73		0.72		-	
	Prediction error (MSE)	213.45		306.95		248.37	
	RMSE	14.61		17.52		15.76	
**SSI^f^**							
	Pseudo R^2^: McFadden	0.17		0.20		-	
	Pseudo R^2^: Cox and Snell (maximum likelihood)	0.54		0.62		-	
	Pseudo R^2^: Nagelkerke (Cragg and Uhler)	0.54		0.62		-	
	Prediction error (MSE)	367.87		632.01		334.15	
	RMSE	19.18		25.14		18.28	
**BSI^g^**							
	Pseudo R^2^: McFadden	0.17		0.14		-	
	Pseudo R^2^: Cox and Snell (maximum likelihood)	0.58		0.55		-	
	Pseudo R^2^: Nagelkerke (Cragg and Uhler)	0.59		0.56		-	
	Prediction error (MSE)	1078.46		102.01		673.4	
	RMSE	32.84		10.1		25.95	

^a^RTI: respiratory tract infection.

^b^Not calculated for the total data set.

^c^MSE: mean square error.

^d^RMSE: root mean square error.

^e^UTI: urinary tract infection.

^f^SSI: surgical site infection.

^g^BSI: bloodstream infection.

### SIR and Corrected SIR by Type of Ward

**Figure 2:**
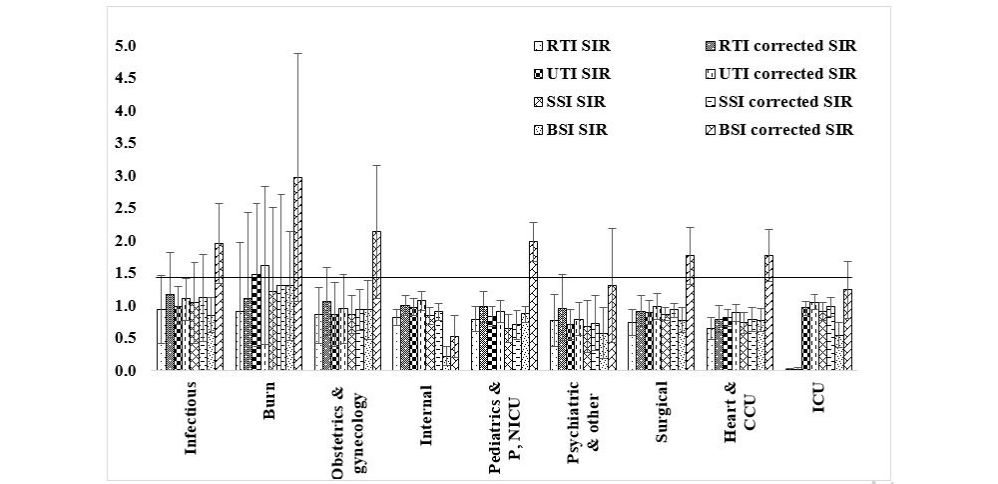
The standardized infection ratio (SIR) and corrected SIR by ward type in Iran, 2018. BSI: bloodstream infection; CCU: cardiac care unit; ICU: intensive care unit; P,NICU: pediatric or neonatal intensive care unit; RTI: respiratory tract infection; SSI: surgical site infection; UTI: urinary tract infection.

The SIR for respiratory tract infections (RTIs; ie, ventilator-associated events, pneumonia events and lower respiratory tract infections, urinary tract infections [UTIs], surgical site infections [SSIs], and bloodstream infections [BSIs]) were 0.024 (95% CI 0-0.071), 0.93 (95% CI 0.86-0.99), 0.86 (95% CI 0.79-0.93), and 0.4 (95% CI 0.23-0.56), respectively. In addition, the corrected SIRs for RTI, UTI, SSI, and BSI were 0.03 (95% CI 0-0.09), 1.02 (95% CI 0.95-1.09), 0.93 (95% CI 0.85-1.007), and 0.91 (95% CI 0.54-1.28), respectively. Also, the corrected SIRs for RTI in the infectious disease, burn, obstetrics and gynecology, and internal medicine wards; for UTI in the burn, infectious disease, internal medicine, and intensive care unit wards; for SSI in the burn and infectious disease wards; and for BSI in most wards were >1. These findings showed that the number of observed HAIs was more than expected (Figure 2).

### SIR and Corrected SIR by Other Variables

The corrected SIR was >1 for accident and burn hospitals, general hospitals, and for-profit hospitals, as well as hospitals with excellent or grade 2/3 accreditations. Considering hospital expertise, the highest SIR and highest corrected SIR were observed in accident and burn hospitals for RTI, UTI, and BSI and in pediatric hospitals for SSI. Also, the highest SIR and highest corrected SIR by hospital affiliation were observed in for-profit hospitals for RTI, government hospitals for UTI, and semigovernment hospitals for BSI. In terms of the type of hospital, the highest SIR and highest corrected SIR were related to nonteaching hospitals for RTI, SSI, and BSI and teaching hospitals for UTI (Table 2).

**Table 2:** Standardized infection ratio (SIR) and corrected SIR by different variables in Iranian hospitals in 2018.

Variables			Hospital number		RTI^a^						UTI^b^					SSI^c^					BSI^d^		
					SIR (95% CI)			Corrected SIR (95% CI)		SIR (95% CI)			Corrected SIR (95% CI)		SIR (95% CI)			Corrected SIR (95% CI)		SIR (95% CI)			Corrected SIR (95% CI)
National			769		0.31			0.4		0.93			0.99		0.86			0.99		0.4			1.14
**Hospital expertise**																							
	Accident & burn		6		1.05 (0.68-1.41)			1.3 (0.85-1.74)		2.41 (1.52-3.3)			2.65 (1.67-3.62)		1.14 (0-2.49)			1.23 (0-2.67)		1.58 (0.96-2.19)			3.59 (2.18-4.99)
	General		578		0.94 (0.87-1)			1.16 (1.08-1.24)		0.98 (0.91-1.05)			1.07 (0.99-1.15)		0.88 (0.81-0.96)			0.95 (0.87-1.03)		0.34 (0.19-0.48)			0.77 (0.43-1.11)
	Heart		11		0.71 (0.37-1.04)			0.87 (0.45-1.29)		0.57 (0.37-0.78)			0.63 (0.41-0.98)		1.06 (0.51-1.6)			1.13 (0.54-1.72)		0.7 (0.48-0.92)			1.6 (1.11-2.09)
	Pediatrics		11		0.61 (0.42-0.8)			0.76 (0.71-1.72)		0.58 (0.38-0.77)			0.63 (0.42-0.84)		1.19 (0.62-1.76)			1.28 (0.66-1.89)		1.23 (0.91-1.55)			2.79 (20.6-3.52)
	Obstetrics & gynecology		21		0.44 (0-0.89)			0.55 (0-1.09)		0.6 (0.3-0.89)			0.66 (0.33-0.98)		0.67 (0.31-1.03)			0.72 (0.33-1.11)		0.86 (0.57-1.14)			1.95 (1.31-2.6)
	Other^e^		34		0.0003 (0-0.0009)			0.0004 (0-0.001))		0.63 (0.37-0.89)			0.69 (0.41-0.98)		0.39 (0.13-0.65)			0.42 (0.14-0.7)		1.49 (0.79-2.18)			3.39 (1.81-4.97)
**Hospital affiliation**																							
		Government		556		0.02 (0-0.06)	0.025 (0-0.07)		0.98 (0.9-1.05)			1.07 (0.99-1.15)		0.89 (0.81-0.97)			0.96 (0.87-1.05)		0.38 (0.22-0.53)			0.85 (0.49-1.21)	
		Semigovernment/other		78		0.79 (0.62-0.96)	0.98 (0.77-1.19)		0.8 (0.64-0.96)			0.88 (0.7-1.05)		0.76 (0.6-0.93)			0.82 (0.64-1.004)		1.05 (0.8-1.31)			2.39 (1.81-2.98)	
		For-profit		135		0.88 (0.69-1.07)	1.09 (0.86-1.33)		0.67 (0.57-0.77)			0.73 (0.62-0.84)		0.73 (0.56-0.89)			0.78 (0.6-0.95)		0.89 (0.71-1.07)			2.03 (1.61-2.45)	
**Accreditation**																							
		Excellent		20		0.72 (0.46-0.98)	0.89 (0.56-1.22)		1.07 (0.84-1.3)			1.17 (0.92-1.43)		0.65 (0.49-0.81)			0.7 (0.52-0.87)		1.14 (0.75-1.52)			2.59 (1.71-3.47)	
		Grade 1		541		0.021 (0-0.062)	0.026 (0-0.077)		0.96 (0.89-1.03)			1.06 (0.98-1.13)		0.87 (0.79-0.96)			0.94 (0.85-1.02)		0.36 (0.21-0.52)			0.83 (0.47-1.18)	
		Grade 2/3		99		0.79 (0.62-0.98)	0.98 (0.77-1.19)		0.58 (0.43-0.73)			0.64 (0.47-1.43)		0.99 (0.8-1.18)			1.06 (0.85-1.27)		0.57 (0.39-0.74)			1.29 (0.89-1.69)	
**Hospital type**																							
		Nonteaching		197		0.89 (0.81-0.97)	1.1 (1.004-1.2)		0.74 (0.66-0.82)			0.81 (0.73-0.9)		0.93 (0.81-1.06)			1.002 (0.87-1.13)		0.69 (0.6-0.78)			1.56 (1.36-1.76)	
		Teaching		465		0.014 (0-0.043)	0.02 (0-0.053)		1.13 (1.03-1.23)			1.24 (1.13-1.35)		0.8 (0.72-0.89)			0.86 (0.78-0.95)		0.36 (0.19-0.53)			0.82 (0.45-1.2)	

^a^RTI: respiratory tract infection.

^b^UTI: urinary tract infection.

^c^SSI: surgical site infection.

^d^BSI: bloodstream infection.

^e^Orthopedic, surgery, cancer, psychiatric.

## Discussion

### Principal Findings

HAIs lead to longer hospital LOS and increased costs for patients and the health care system. Knowing the reasons for, type of, and rate of HAIs can be very helpful for optimal management and improving service quality. Therefore, the establishment of a nosocomial infection control committee, implementation of educational programs, attention to the physical structure of hospital wards, and providing motivational and attitudinal mechanisms in infection control are important factors that can reduce HAIs [14]. Based on the results, the type of ward, hospital affiliation, the mean age of patients, male-to-female patient ratio, number of device-related infections, number of catheter-related infections, surgery-to-surgery bed ratio, death-to-bedridden ratio, and ventilator-day to catheter-day ratio were the predictor variables for HAIs.

In a study in Liguria, age >54 years, LOS, surgery, exposure to more devices, and exposure to vascular catheters were confirmed as factors associated with HAIs in a multivariable analysis. However, the type of ward and the size of the hospital (number of beds) had no relationship with the occurrence of HAIs [15].

Implementing infection prevention and control activities is critical for reducing the burden of HAIs and should be tailored to local needs. These standards are far from the goals of the infection prevention guidelines in the hospitals included in this study, which recommend that there should be one full-time infection control nurse for every 100 beds in acute care hospitals and every 150 beds in long-term acute care hospitals or every 250 beds, as defined by the WHO guidelines [16,17]. It is also important to note that staffing has been identified as a human resource for infection prevention [18].

In this study, the corrected SIR based on observed and predicted infections for RTI, UTI, SSI, and BSI were 0.03, 1.02, 0.93, and 0.91, respectively. Also, the corrected SIR for RTI in the infectious disease, burn, obstetrics and gynecology, and internal medicine wards; for UTI in the burn, infectious disease, internal medicine, and intensive care unit wards; for SSI in the burn and infectious disease wards; and for BSI in most wards were >1, indicating that the observed number of HAIs was more than expected. This index was >1 for accident and burn hospitals, general hospitals, and for-profit hospitals, as well as for hospitals with excellent or grade 2/3 accreditations.

Since the highest SIR was related to UTI, prevention strategies for reducing the risk of these infections, such as educating health care personnel regarding the indications for catheter use, proper procedures for the insertion and maintenance of catheters, appropriate infection control measures to prevent catheter-related infections, hand hygiene, and aseptic procedures, should be considered [19,20].

Improving hand hygiene, reducing and avoiding unnecessary urinary catheters, placing urinary catheters using an aseptic method and keeping them according to the instructions, determining the need for a urinary catheter on a daily basis and removing it as soon as possible, and managing an incontinence catheter should be considered to reduce these infections if possible.

In a study by Martillo et al [21], there were 102 central line–associated BIs (CLABSIs) and 58,321 line-days in 2017. The CLABSI rate was 1.75 infections per 1000 days, and the SIR was 1.25. Also, in 2018, the number of CLABSIs decreased by 58% (59 infections and 56,893 line-days). The CLABSI rate was 1.03 infections per 1000 days, and the SIR was 0.91. In 2017, 58,621 central line-days were utilized across the hospital with a standardized utilization ratio of 0.73, while in 2018, there were 56,893 central line-days with a standardized utilization ratio of 0.81 [21]. Therefore, based on the results, the use of a specialized team such as the comprehensive vascular access service (VAS) team ensures adherence to the best practices during catheter insertion and enhances nurse training for device maintenance, especially in patients who are at risk for complications. Reports have shown that using dedicated VAS teams seems to be a good and cost-effective strategy, as these teams are associated with greater success for first-time insertion and thus improve efficiency and safety and reduce side effects [21,22]. Another report by Brunelle [23] showed that a team dedicated to the maintenance of central venous catheters reduces BSI from 45 to 19 infections per year. On the other hand, a Cochrane systematic review that attempted to assess the role of specialized teams in device insertion failed to find clinical trials that supported the role of these teams compared with general practitioners [24].

In the study by Słowik et al [25], the SIR did not exceed 1, and the incidence of SSI after hip arthroplasties was at a level comparable to that of European countries (0.7 for patients without a risk factor, 0.8 for patients with 1 risk factor, and 0.3 for patients with 2 or 3 risk factors). The SIR for SSIs in knee arthroplasties exceeded 1 at all 3 levels and was obtained as follows: 7 for patients without risk factors, 2 for patients with 1 risk factor, and 2 for patients with 2 or 3 risk factors [25].

In a study in Italy, the prevalence of observed infections in 13 of 18 hospitals was lower than expected, while in 4 hospitals, the SIR was >1. One hospital had reached 4.9, which means a 390% increase in the observed infections compared with the expected number [15]. Boev et al [26], in an investigation using the results of various studies, reported the SIRs for CLABSI, catheter-associated UTI, and SSI (colon surgery only) to be 5, 1, and 0.98, respectively.

### Strengths and Limitations

Conventional epidemiological indicators do not have the necessary efficiency to prioritize programs. Thus, new indicators were introduced, including the SIR, which corrects the prediction of the expected nosocomial infections by considering the variables affecting this rate. It can be said that one of the strengths of this study is to address this index and calculate it. Another strength of the study is the use of more than one source, namely INIS and AVAB; the use of registered data for a multicenter, comprehensive, and national-level study to more accurately estimate the HAIs in Iran; and the use of the NHSN method, which is considered less frequently by researchers. One other advantage of the study is the calculation of the corrected SIR.

On the other hand, the following factors can be considered as the study limitations: lack of a complete and comprehensive data source for HAIs and hospital-related variables; problems related to the data including underreporting, registration of false negatives, missing data, problems related to the definition of infections, and insufficient quality control of data, among others; failure to collect data from all hospitals in Iran; lack of new and similar studies in Iran that can be compared with the current study (the results were compared with those of only a few studies, which were not performed with the same method as this study); failure to calculate the SIR for SSIs according to the type of procedure; and the possibility of some biases due to the retrospective study. However, as mentioned earlier, in this study, to overcome the limitations of data sources and data problems, several sources as well as the NHSN codes were used to estimate HAIs more accurately and completely. Also, regarding the limitation of not collecting data from all hospitals in Iran, it can be said that this study was performed using recorded data with a coverage of about 75%, which were randomly collected from all the provinces in the country; therefore, it can be claimed that the results are reliable.

### Conclusions

The results of this study can help to promote preventive measures based on scientific evidence. They can also lead to the continuous improvement of the monitoring system by collecting and systematically analyzing data on HAIs. Addressing HAIs in low-resource countries may require significant investment and commitment to an infection prevention and control program, which includes training and deployment of infection control care professionals, as an additional strategy to help implement the guidelines. As a result, although significant progress has been made in recent years in implementing prevention strategies in Iranian hospitals, HAI remains a public health concern. The higher observed number of HAIs than expected, especially for UTI, indicates the need for a nosocomial infection care system and effective policies for identifying the types of HAIs and the related factors and encourages the hospitals to better control their infection rates by establishing a benchmarking system.

## Abbreviations

AVAB: hospital statistics and information system
BOR: bed occupancy rate
BSI: bloodstream infection
BTO: bed turnover
CDC: Centers for Disease Control and Prevention
CF: correction factor
CLABSI: catheter line–associated bloodstream infection
HAI: hospital-acquired infection
INIS: Iranian Nosocomial Infections Surveillance System
LOS: length of stay
MSE: mean square error
NHSN: National Healthcare Safety Network
NNIS: National Nosocomial Infections Surveillance
RMSE: root mean square error
RTI: respiratory tract infection
SIR: standardized infection ratio
SSI: surgical site infection
UTI: urinary tract infection
VAS: vascular access service
